# Constitutive glucose dehydrogenase elevates intracellular NADPH levels and luciferase luminescence in *Bacillus subtilis*

**DOI:** 10.1186/s12934-022-01993-0

**Published:** 2022-12-20

**Authors:** Yuzheng Wu, Honami Kawabata, Kyosuke Kita, Shu Ishikawa, Kan Tanaka, Ken-ichi Yoshida

**Affiliations:** 1 Department of Science, Technology and Innovation, Kobe University, 1-1 Rokkodai, Nada, Kobe, 657 8501 Japan; 2 Laboratory for Chemistry and Life Science, Institute of Innovative Research, Tokyo Institute of Technology, Tokyo, Japan; 3 Core Research for Evolutional Science and Technology, Japan Science and Technology Agency, Tokyo, Japan

**Keywords:** *Bacillus subtilis*, Inositol, NADPH, Luciferase

## Abstract

**Background:**

Genetic modifications in *Bacillus subtilis* have allowed the conversion of *myo*-inositol into *scyllo*-inositol, which is proposed as a therapeutic agent for Alzheimer’s disease. This conversion comprises two reactions catalyzed by two distinct inositol dehydrogenases, IolG and IolW. The IolW-mediated reaction requires the intracellular regeneration of NADPH, and there appears to be a limit to the endogenous supply of NADPH, which may be one of the rate-determining factors for the conversion of inositol. The primary mechanism of NADPH regeneration in this bacterium remains unclear.

**Results:**

The *gdh* gene of *B. subtilis* encodes a sporulation-specific glucose dehydrogenase that can use NADP^+^ as a cofactor. When *gdh* was modified to be constitutively expressed, the intracellular NADPH level was elevated, increasing the conversion of inositol. In addition, the bacterial luciferase derived from *Photorhabdus luminescens* became more luminescent in cells in liquid culture and colonies on culture plates.

**Conclusion:**

The results indicated that the luminescence of luciferase was representative of intracellular NADPH levels. Luciferase can therefore be employed to screen for mutations in genes involved in NADPH regeneration in *B. subtilis*, and artificial manipulation to enhance NADPH regeneration can promote the production of substances such as *scyllo*-inositol.

## Background

Efficient metabolic engineering of microorganisms often requires the consideration of strategies to supply sufficient cofactors for the oxidation–reduction reaction. For example, when central carbon metabolism was manipulated to increase the supply of NADPH, *Escherichia coli* produced xylitol [1]. In addition, when endogenous and heterogenous genes for NADPH regeneration were overexpressed, γ-polyglutamic acid synthesis of *Bacillus licheniformis* was improved [2].

**Fig. 1:**
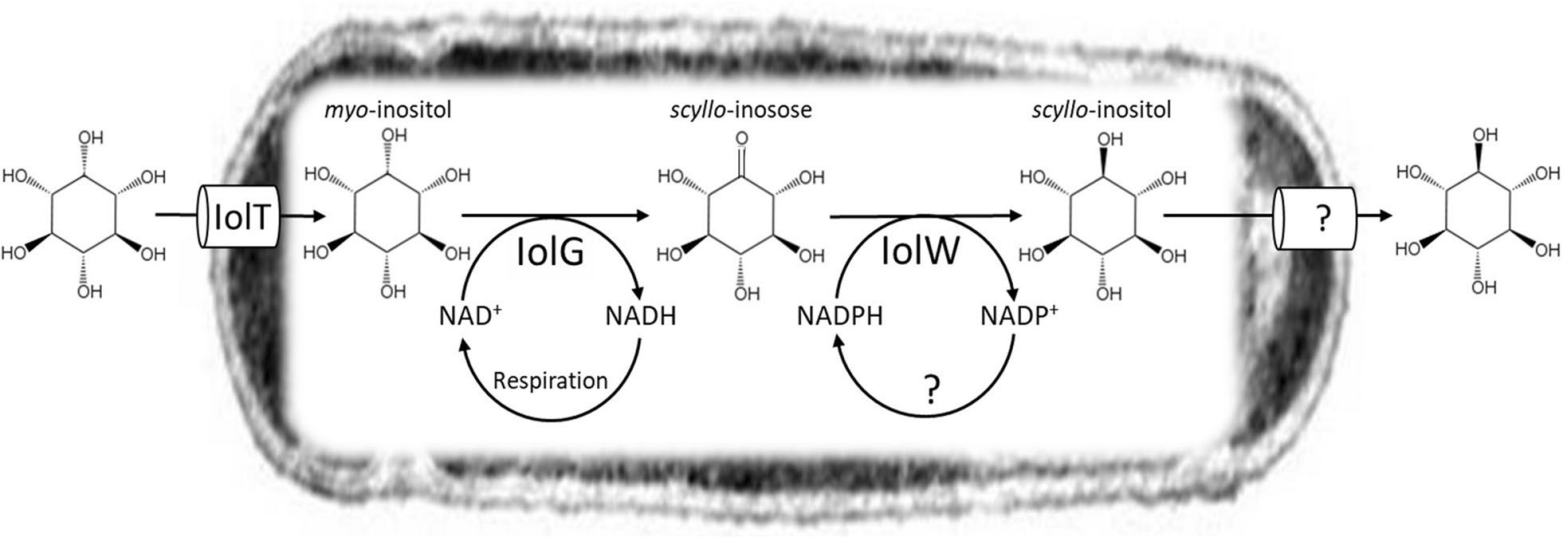
A diagram illustrating how IolG and IolW function in the *B. subtilis* cell factory producing *scyllo*-inositol. In the *B. subtilis* cell factory, the IolG- and IolW-mediated reactions are coupled to convert *myo*-inositol to *scyllo*-inositol depending on NAD^+^ and NADPH, respectively

We have studied the practice of metabolic engineering in *Bacillus subtilis* with the aim of promoting research and development to modify its inositol metabolism to produce the desired chemicals. Nine stereoisomers of inositol (cyclohexanehexol) are known, which are epimers of the six hydroxyl groups. *myo*-Inositol is the most abundant isomer in nature (*cis*-1,2,3,5-*trans*-4,6-cyclohexanehexol); the other stereoisomers are rare and thus expensive. Interestingly, some rare inositol stereoisomers exert remarkable biological activities. For example, *scyllo*-inositol is a promising drug candidate for the treatment of Alzheimer’s disease, because it has chemical chaperone activity, which has been shown to inhibit the aggregation of abnormal proteins associated with Alzheimer's disease and reduce neurocytotoxicity and cognitive impairment [3–7]. By modifying the specific metabolic pathway for inositol catabolism [8], *B. subtilis* cell factories able to convert *myo*-inositol into *scyllo*-inositol have been produced (Fig. 1) [9–11]. The conversion takes place by selectively combining two reactions catalyzed by IolG and IolW. As IolG is an NAD^+^-dependent *myo*-inositol dehydrogenase for the conversion of *myo*-inositol into *scyllo*-inosose, the IolG reaction reduces NAD^+^ to NADH. In contrast, IolW is an NADP^+^-dependent *scyllo*-inositol dehydrogenase for the conversion of *scyllo*-inosose into *scyllo*-inositol with oxidization of NADPH into NADP^+^. Therefore, the overall bioconversion depends on the regeneration of both NAD^+^ and NADPH. Although the mechanism is still unclear, the *scyllo*-inositol produced within *B. subtilis* cells is secreted and accumulates in the medium [9–11]. For this bioconversion, the cell factories were maintained under aerobic conditions. Accordingly, it is unlikely that the NAD^+^ supply would be insufficient as NADH is preferentially oxidized into NAD^+^ via the respiratory chain. In contrast, to support the second reaction, some reactions to reduce NADP^+^ and regenerate NADPH had to be enhanced as required. It was suggested that there may be a limitation on the ability of *B. subtilis* to regenerate NADPH, which may restrict the performance of the cell factories for the production of *scyllo*-inositol [11].

In *E. coli* and many other bacterial species, NAD(P)^+^ transhydrogenases use NADH to reduce NADP^+^ to NADPH [12]. However, in *B. subtilis*, no typical gene for this enzyme has been deduced within its genome, although the possible existence of NAD(P)^+^ transhydrogenase activity has been reported [13, 14]. When *E. coli pntAB*, encoding the NAD(P)^+^ transhydrogenase, was expressed in the *B. subtilis* cell factory converting *myo*-inositol into *scyllo*-inositol, the conversion was slightly enhanced [11]. The results suggested that the *E. coli* NAD(P)^+^ transhydrogenase could elevate NADPH levels to improve the efficiency of *scyllo*-inositol production by enhancing the IolW reaction.

However, the primary mechanism for endogenous NADPH regeneration in *B. subtilis* remains unclear. As a minimum, we can presume that some reactions in central metabolic pathways, such as glycogenesis, pentose phosphate shunt, and the TCA cycle, could act to regenerate NADPH. In glycogenesis, one of the malic enzymes encoded by *ytsJ* could regenerate NADPH [15]. In the reaction following this, glyceraldehyde 3-phosphate dehydrogenase encoded by *gapB* consumes NADPH. Therefore, it is unlikely that NADPH is obtained from glycogenesis [16]. In contrast, in the pentose phosphate shunt, two reactions can provide NADPH; one is catalyzed by Zwf (glucose 6-phosphate dehydrogenase) [17] and the other is catalyzed by GndA (6-phosphogluconate dehydrogenase) [18]. In addition, in the TCA cycle, Icd (isocitrate decarboxylase) is also able to regenerate NADPH [19]. Among the enzymes for these reactions involving the regeneration of NADPH, CcpA and CcpC regulate the expression of Icd under carbon catabolite repression [20, 21], and *ytsJ* is controlled by LexA and induced by DNA damage [22]. However, no regulator is reported for either Zwf or GndA as both are constitutively expressed [23, 24]. In any case, in *B. subtilis*, it remains unclear if the function/expression of any specific enzyme capable of regenerating NADPH, including those mentioned above, could be enhanced by the counter-reaction of the increased consumption of NADPH produced by metabolic engineering.

Bacterial luciferase systems are dependent on a cascade reaction, which is catalyzed by a series of enzymes encoded by the *luxABCDE* operon. LuxA and LuxB are the α- and β-subunits of the luciferase, respectively. LuxC, LuxD, and LuxE constitute the fatty acid reductase complex that synthesizes the long-chain fatty aldehyde, consuming NADPH and ATP. The luciferase reaction is an oxidative process in which molecular oxygen oxidizes FMNH_2_ and the long-chain fatty aldehyde to the corresponding acid. The reaction forms an excited intermediate, which is dehydrated to FMN and emits blue–green light [25]. NAD(P)H-dependent FMN reductases occur naturally in most bacterial species, including *E. coli* and *B. subtilis*; thus, FMN is readily reduced into FMNH_2_ depending on the available NAD(P)H. Therefore, the bioluminescence of bacterial luciferase is dependent on NAD(P)H. Recently, a bacterial luciferase was introduced into *E. coli* to act as a reporter for the redox state of cells, representing the intracellular levels of NAD(P)H [26].

In this study, glucose dehydrogenase, which is encoded by *gdh* and originally expressed only during sporulation with NAD(P)H regeneration, was overexpressed in one of the *B. subtilis* cell factories converting *myo*-inositol into *scyllo*-inositol. It was expected that the thus regenerated NADPH might enhance the conversion of inositol, especially the second step reaction catalyzed by IolW. In addition, we also introduced *luxABCDE* of *Photorhabdus luminescens* [27] to examine the potential to monitor the intracellular levels of NAD(P)H by the change in luciferase luminescence. The efficiency of inositol conversion was analyzed in parallel to the luciferase luminescence, which could serve as a nondestructive means to report NADPH levels in vivo.

## Results

### Introduction of *gdh* for artificial regeneration of NADPH in *B. subtilis*

*B. subtilis* does not appear to possess a gene for a typical NAD(P)^+^ transhydrogenase within its genome [13, 14]. Previously, *E. coli pntAB* encoding the NAD(P)^+^ transhydrogenase was expressed in the *B. subtilis* cell factory converting *myo*-inositol into *scyllo*-inositol. The conversion was slightly enhanced, indicating that the elevated NADPH levels could contribute to an increased efficiency of *scyllo*-inositol production, facilitating the IolW-mediated reaction [11]. However, this regeneration of NADPH is dependent on the available NADH and is unlikely to be efficient owing to the competition for NADH with the efficient respiratory chain production of ATP.

**Fig. 2:**
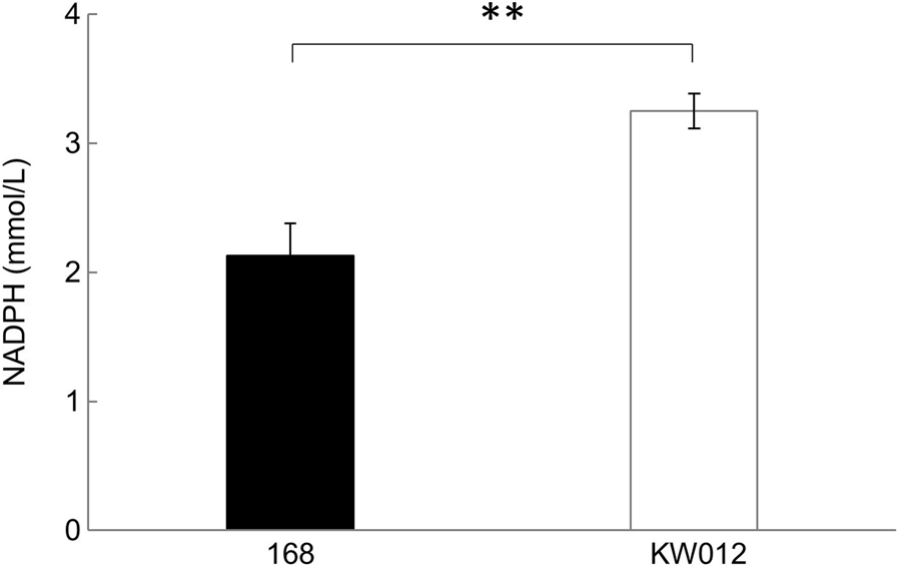
Intracellular levels of NADPH in 168 and KW012. Strains 168 and KW012 were grown for 24 h, and the cells were harvested to determine the intracellular levels of NADPH. The experiments were repeated three times; similar results were obtained and the results are presented as mean values ± SD (**, *p* < 0.01)

*B. subtilis gdh* encodes a glucose dehydrogenase for the conversion of D-glucose into D-glucono-1,5-lactone with the generation of NAD(P)H [28]. As the transcription of *B. subtilis gdh* depends on SigG, it acts only within the forespore during sporulation [29]. *B. subtilis gdh* was expressed in *E. coli*, which promoted NADPH regeneration and efficiently stimulated the NADPH-dependent ketoreductase reaction in permeabilized cells [30]. In this study, *gdh* was expressed artificially under the transcriptional control of a constitutive P*spac* promoter. Two strains, 168 and KW012 [*aprE*::(P*spac-gdh spc*)] (Table 1), were grown in 4% Soytone medium for 24 h. The crude extracts of the harvested cells were used to measure the NADPH levels (Fig. 2). The NADPH concentration in 168 was 2.13 mM, whereas that in KW012 was 3.25 mM, indicating a 1.5-fold increase in the intracellular NADPH concentration with a significant difference upon the artificial expression of *gdh*.

**Table 1:** Bacterial strains used in this study

Strain	Genotype	Source or reference
*B. subtilis*		
168	*trpC2*	Laboratory stock
KU302	*amyE*::(P*rpsO-iolG-iolW-iolT cat*) ∆*iolABCDEF* Δ*iolHIJ* Δ*iolX* Δ*iolR trpC2*	[11]
KW012	*aprE*::(P*spac-gdh spc*) *trpC2*	This study
KW018	*aprE*::(P*spac-gdh spc*) in the KU302 background	This study
KW028	*sacA*::(P*yhaM-luxABCDE erm*) in the KW018 background	This study
YG010	*sacA*::(*luxABCDE cat*) *trpC2*	This study
WY002	*sacA*::(P*yhaM-luxABCDE erm*) in the KU302 background	This study

**Fig. 3:**
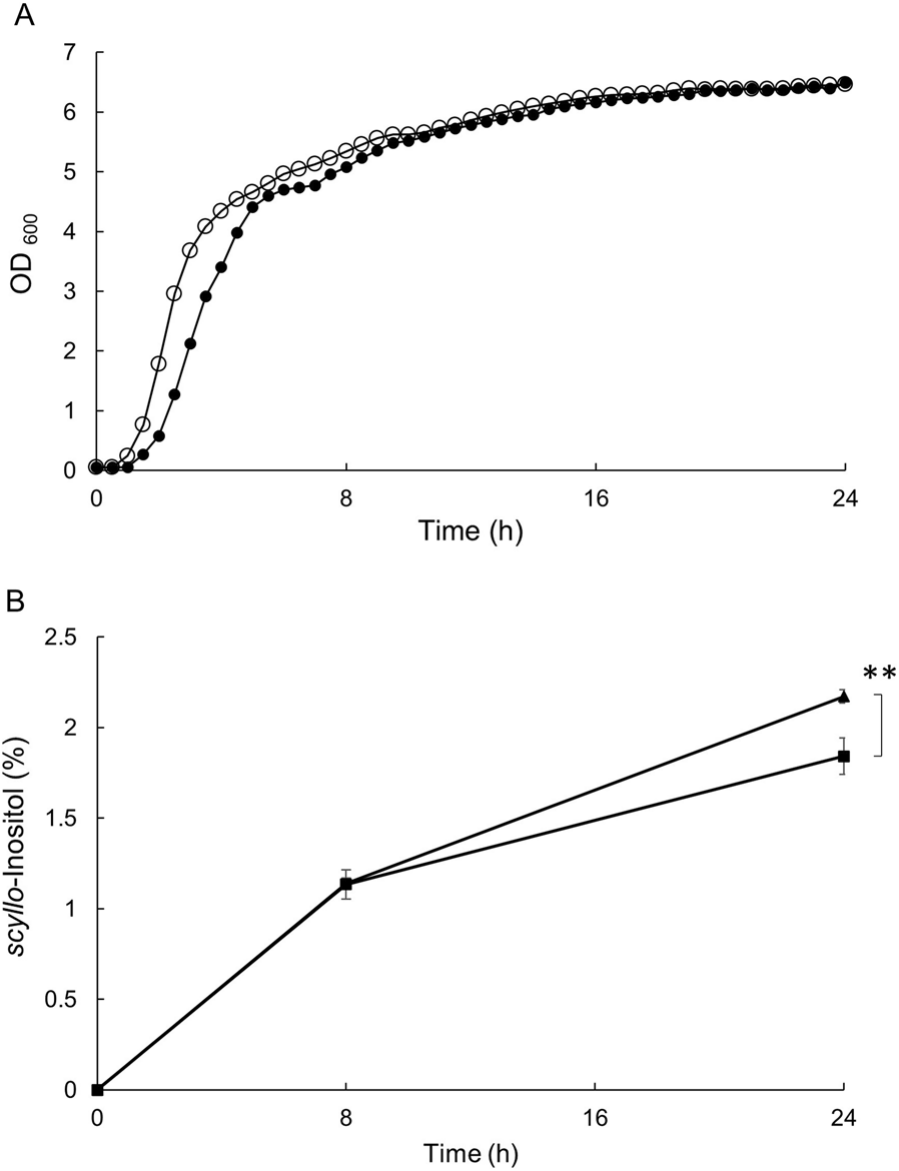
*scyllo*-Inositol production in KU302 and KW018. Strains KU302 (closed circles) and KW018 (open circles) were grown for 24 h to convert *myo*-inositol into *scyllo*-inositol. Growth curves (**A**) and concentrations (%) of *scyllo*-inositol in the culture medium were measured at 0, 8, and 24 h (**B**). The experiments were repeated three times; similar results were obtained. A set of representative growth curves is shown (**A**) and *scyllo*-inositol concentrations are presented as mean values ± SD (**, *p* < 0.01) (**B**)

Next, the constitutive expression of *gdh* was introduced into the strain converting *myo*-inositol into *scyllo*-inositol [11]. Strains KU302 [*amyE*::(P*rpsO-iolGWT cat*) ∆*iolABCDEF* ∆*iolHIJ* ∆*iolX* ∆*iolR*] and KW018 [*aprE*::(P*spac-gdh spc*) in KU302 background] (Table 1) were inoculated into 4% Soytone medium supplemented with 50 g/L *myo*-inositol and allowed to grow at 37 °C with shaking. The two strains showed that the growth curves were essentially similar to each other, although KW018 had a slightly shorter lag phase (Fig. 3A). Samples of culture medium were collected at 0, 8, and 24 h after inoculation and analyzed to measure the level of inositol stereoisomers (Fig. 3B). KU302 and KW018 produced almost the same amount (12 g/L) of *scyllo*-inositol over 8 h, and 18 and 22 g/L over 24 h, respectively. The increase in *scyllo*-inositol production in KW018 may be because the constitutive expression of *gdh* elevates the intracellular NADPH levels over 24 h.

### Introduction of *luxABCDE* to monitor the intracellular NAD(P)H levels

**Fig. 4:**
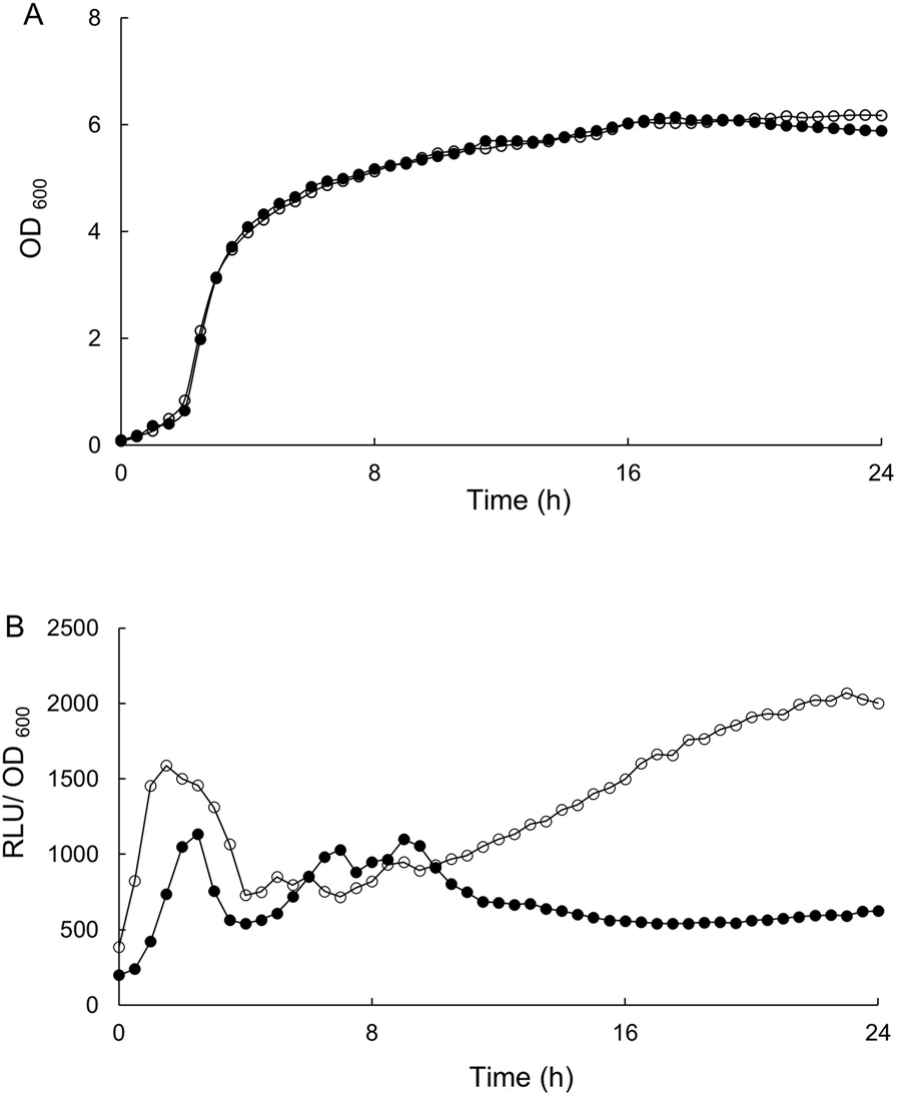
Luciferase luminescence in liquid cultures of WY002 and KW028. Strains WY002 (closed circles) and KW028 (open circles) were cultivated in a microtiter plate for 24 h. Growth curves (**A**) and RLU normalized by OD_600_ for the cells (**B**). The experiments were repeated at least three times; similar results were obtained, and a representative set of data is presented

Recently, a gene set for the bacterial luciferase system was introduced into *E. coli* as a reporter to monitor the intracellular redox state [26]. We adapted the *luxABCDE* operon encoding the luciferase system of *P. luciferase* [27] under the control of a constitutive P*yhaM* promoter [31]. Two *B. subtilis* strains were constructed: WY002 [*sacA*::(P*yhaM*-*luxABCDE erm*) in the KU302 background] and KW028 [*aprE*::(P*spac*-*gdh spc*) *sacA*::(P*yhaM*-*luxABCDE erm*) in the KU302 background] (Table 1). The two strains were inoculated into 4% Soytone medium and allowed to grow at 37 °C in a microtiter plate with shaking; the luminescence of luciferase and the optical density for cells at 600 nm (OD_600_) were monitored continuously for 24 h (Fig. 4). The two strains had almost similar growth patterns. In contrast, the luminescence of luciferase normalized by OD_600_ in WY002 exhibited several small peaks during the logarithmic growth and remained relatively constant during the stationary phase, whereas that in KW028 resulted in a broad peak higher than in WY002 during the early logarithmic growth phrase and the subsequent gradual increase up to levels almost three times higher than in WY002 over 24 h. The results indicated that the artificial expression of *gdh* elevated the luminescence of luciferase first in the logarithmic growth phase and it accumulated later during the stationary phase, which could coincide with the higher concentration of NADPH found in KW012 after 24 h.

**Fig. 5:**
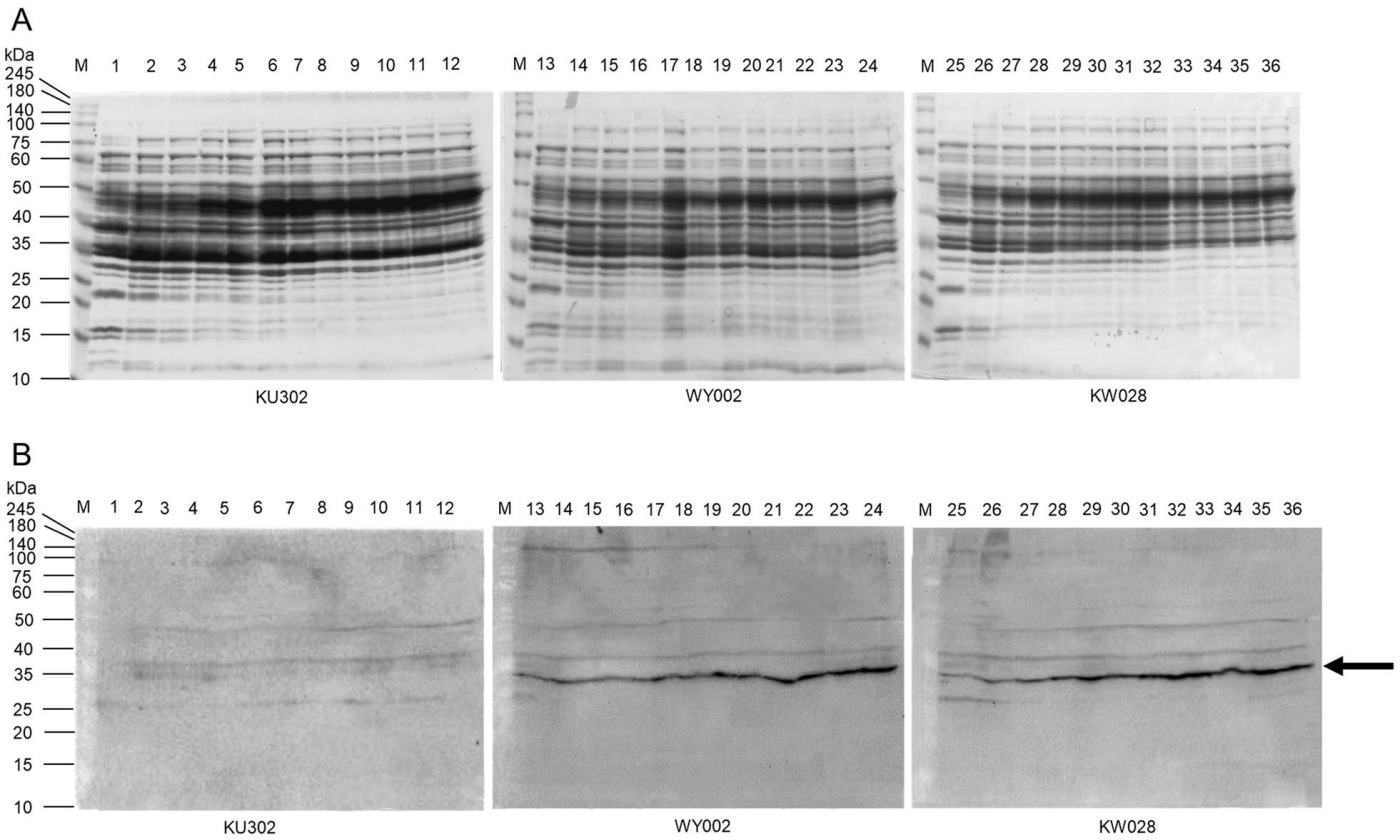
Constant production of LuxB protein in WY002 and KW028. Strains KU302, WY002, and KW028were were grown for 24 h. Cells solutions of 1 OD_600_ unit was harvested every 2 h and subjected to 12% SDS-PAGE; lanes 1–12, 13–24, and 25–36 corresponding to 2–24 h cultures of KU302, WY002, and KW028, respectively. Lane M contained size marker proteins. The images of CBB-stained gel (**A**) and corresponding western blotting for LuxB protein (**B**). An arrowhead on the right indicates the position of LuxB. The experiments were repeated at least three times with similar results, and a representative set of data is presented

To confirm that the difference in luciferase luminescence was not due to differences in its expression level, one subunit of luciferase (LuxB protein) in cell extracts of the two strains was detected over time by western blotting using a custom-made rabbit anti-LuxB polyclonal antibody (Fig. 5). No specific band indicating the presence of LuxB protein was detected in the negative control strain KU302, which does not possess the luciferase operon. In contrast, the same specific bands for LuxB were detected almost uniformly in both WY002 and KW028, with no significant difference observed between the two strains.

### Artificial expression of *gdh* enhanced luciferase luminescence in individual colonies cultured on plates

**Fig. 6:**
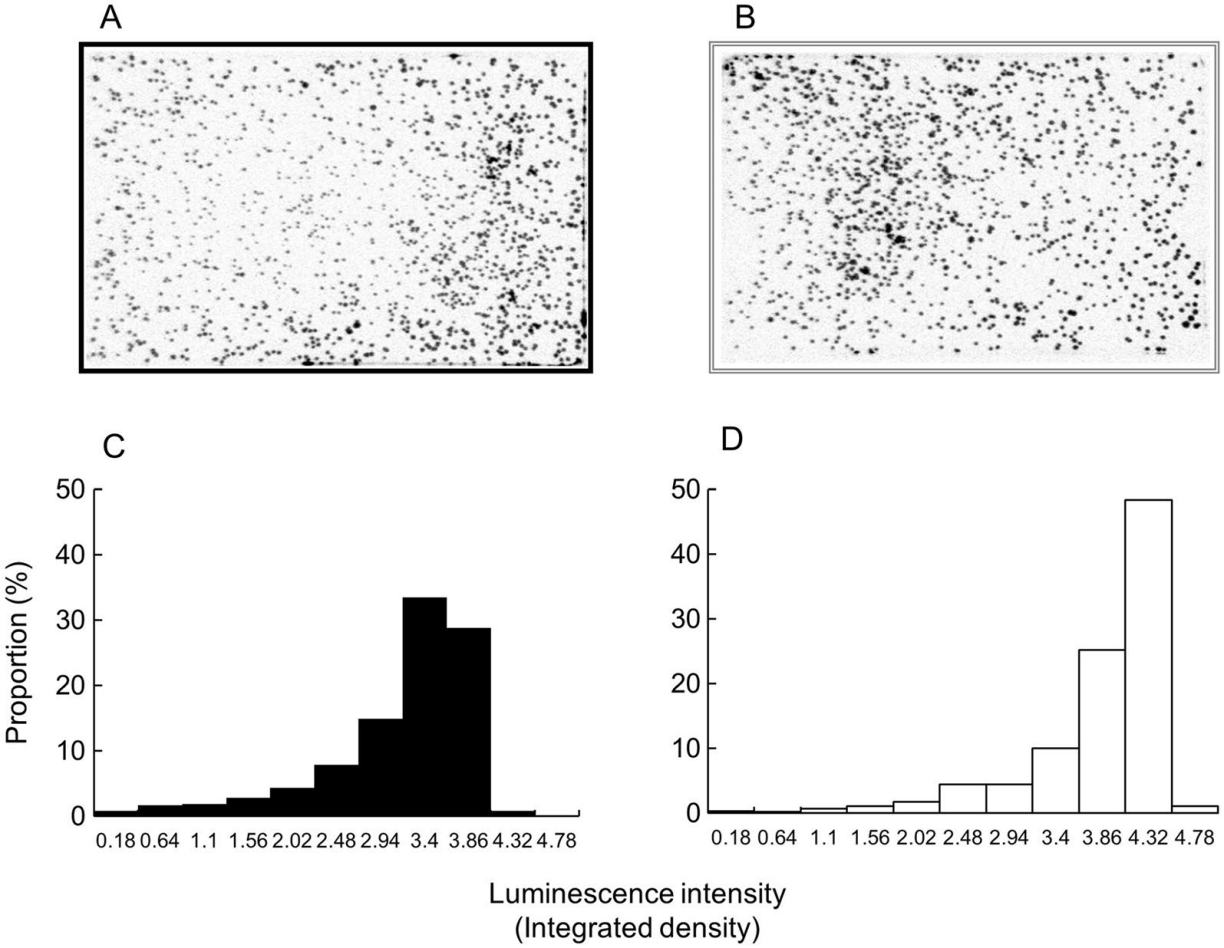
Luciferase luminescence in WY002 and KW028 colonies. Culture plates containing numerous colonies of WY002 (**A**) and KW028 (**B**) were observed to generate the luminescence reversal images. The images were analyzed to quantify the luminescence intensity (integrated density) of each of the colonies and histograms were drawn to compare WY002 (**C**) and KW028 (**D**) for the proportion (percentage) of colonies with different luminescence intensity. The experiments were repeated at least three times; similar results were obtained, and a representative set of data is presented

The two strains, WY002 and KW028, were spread on 4% Soytone medium plates and allowed to form 500–1,000 colonies at 37 °C. After incubation for 11 h, the reversal image of the entire plate for luminescence was captured to generate reversal images for luminescence (Fig. 6A, B). The luminescence intensity of each colony was quantified and histogram analysis was performed to compare WY002 and KW028 for the distribution of colonies with different luminescence intensities (Fig. 6C, D). The colony distribution of WY002 and KW028 revealed peaks at luminescence intensity windows of 3.4–3.86 and 4.32–4.78, respectively. Notably, no less than 50% of the KW028 colonies had stronger luminescence intensities than those of WY002. The results suggested that KW028 and WY002 could be distinguished by the difference in luminescence intensity of the colonies.

## Discussion

*B. subtilis gdh* can react with D-glucose or 2-deoxy-D-glucose using NAD^+^ or NADP^+^ as a cofactor [28, 29]. Its expression is SigG-dependent and under the control of SpoVT, which strictly limits its expression only within the forespore during sporulation [32]. In this study, we artificially expressed *gdh* under the control of a constitutive promoter in cells during the logarithmic and stationary growth phases. Consequently, the biochemical assay indicated the increase in the intracellular concentration of NADPH with a significant difference (Fig. 2). It is thought that the intracellular NADPH concentration is usually maintained at higher levels, but we confirmed that this enzyme caused further elevation, especially in the latter stationary growth phase. It may be difficult to utilize the other coenzyme, NAD^+^, because of competition with many other enzymes in the glycolytic pathway, TCA cycle, and so on.

In contrast, when the artificial conversion of *myo*-inositol to *scyllo*-inositol (Fig. 1) was combined with the constitutive expression of *gdh*, the increased NADPH described above might contribute to increase production of *scyllo*-inositol during the later stationary growth phase, presumably owing to the reduction of *scyllo*-inosose by IolW that was dependent on NADPH [10]. This was at a level no less than the effect of our previous result with the introduction of transhydrogenase [11]. The reduction of NADP^+^ by transhydrogenase requires NADH, and this enzyme is primarily presumed to compete for NADH with the respiratory chain. Because there is no such competition for Gdh, we expected that Gdh could more efficiently increase the production of *scyllo*-inositol. However, the main product of the Gdh reaction, D-glucono-1,5-lactone, may also be flushed into the pentose phosphate pathway more than usual, possibly reducing the supply of glucose to glycolysis. Furthermore, the Entner–Doudoroff pathway is not thought to occur in *B. subtilis* [33], and the influx of carbon sources into the pentose phosphate pathway may be extremely biased, resulting less pyruvate to feed into the TCA cycle for energy production. Thus, such an overall imbalance in global metabolism may be a reason for the limited effects of Gdh. As detailed metabolomic analyses are needed to test the hypothesis mentioned above, further studies will be conducted elsewhere.

Based on the finding that the constitutive expression of *gdh* in *B. subtilis* can increase the intracellular levels of NADPH, another important point found in this study is that the luminescence of bacterial luciferases is increased by the enhanced regeneration of NADPH. Bacterial luciferases require NADPH and ATP to obtain fatty acid aldehydes as the substrate and FMNH_2_ as the cofactor to mono-oxygenate fatty aldehydes for the luminescence reaction. In addition, NAD(P)H is required to reduce FMN to obtain FMNH_2_. In other words, NADPH is essential for the reactions involved in the luminescence-generating process of bacterial luciferase; thus, we hypothesize that the intracellular levels of NADPH may affect the luminescence of luciferase. Indeed, this idea was confirmed experimentally in this study. Even in the absence of artificial *gdh* expression, the intensity of luciferase luminescence fluctuated as the growth phase proceeded. A similar phenomenon was observed in *E. coli* [26] and may reflect fluctuations in the intrinsic redox state, i.e., the intracellular concentrations of redox-active cofactors such as NADH and NADPH. In the presence of *gdh* expression, the luminescence intensity of luciferase was clearly increased, especially during the later stationary phases. These results indicate that the luminescence of bacterial luciferase could be used as a nondestructive means to measure intracellular NADPH levels.

Furthermore, changes in luciferase luminescence in response to *gdh* expression were observed not only in liquid cultures but also in colonies grown on agar medium. Introducing bacterial luciferase into a randomly mutagenized library of *B. subtilis* would allow us to select colonies with significantly enhanced or attenuated luminescence. Such colonies are likely formed by mutants with altered gene functions that cause abnormal intracellular NADPH levels. As the mechanism of NADPH regeneration in *B. subtilis* remains unclear, the analysis of such mutants may help us to resolve this mystery. In addition, some of the efficient NADPH-enhancing mutants can also serve as a basis to promote the production of desired substances, depending on the NADPH supply. Moreover, we will use them to improve the *scyllo*-inositol production.

## Conclusions

Intracellular levels of NADPH increased in *B. subtilis* upon constitutive expression of *gdh*, which encodes glucose dehydrogenase, leading to the enhanced conversion of *myo*-inositol to *scyllo*-inositol through the NADP^+^-dependent *scyllo*-inositol dehydrogenase. In addition, the bacterial luciferase derived from *P. luminescens* became more luminescent in the cells both in liquid culture and colonies on culture plates. The results indicated that the luciferase luminescence was representative of the intracellular NADPH levels. Therefore, luciferase can be applied to screen mutations causing intracellular levels of NADPH in *B. subtilis*, which may allow us to elucidate the unknown mechanisms of NADPH regeneration.

## Methods

### Bacterial strains and culture conditions

The strains of *B. subtilis*, listed in Table 1, were maintained on LB agar plates (Difco, Franklin Lakes, NJ) at 37 °C. The culture media were supplemented with antibiotics as required: 0.5 µg/mL erythromycin, 100 µg/mL spectinomycin, 10 µg/mL chloramphenicol, 5 µg/mL tetracycline, and 10 µg/mL kanamycin. For the experimental conditions, precultures of the bacteria were diluted in 50 mL of 4% Soytone medium, composed of 4% Bacto Soytone (Difco), 0.5% Bacto Yeast Extract (Difco), and 0.2% glucose, in a 500 mL baffled flask to an OD_600_ of 0.05 and allowed to grow at 37 °C with shaking at 200 rpm. The medium for inositol conversion contained 5% *myo*-inositol.

### Construction of strains

Strain KW012 [*aprE*::(P*spac-gdh spc*)] was constructed as follows. Two PCR fragments corresponding to *aprE*-front and *aprE*-back were amplified from DNA of 168 with the primer pairs cmp141/cmp195 and cmp199/cmp150 (Table 2), respectively. The *spc*-P*spac* fragment was amplified from the DNA of pDGIEF [34] by PCR with the primer pair cmp12/cmp196 (Table 2). The *gdh* fragment was from DNA of 168 with the primer pair cmp197/cmp198 (Table 2). The *aprE*-front, *spc*-P*spac*, *gdh*, and *aprE*-back fragments were ligated, in this order, by recombinant PCR with the primer pair cmp141/cmp150, as cmp195, cmp196, and cmp198 are given complementary sequences to cmp12, cmp197, and cmp199, respectively. The resulting recombinant PCR fragment was used to transform strain 168 to be spectinomycin-resistant, to yield KW012. Strain KW018 was made from KU302 [11] transformed to be spectinomycin-resistant with DNA of KW012.

**Table 2:** PCR primers used in this study

Primer	Sequence (5ʹ–3ʹ)
cmp12	aaagtaagcacctgttattgc
cmp141	cgcagcatgtttcatttaga
cmp150	tgtctttgcttggcgaat
cmp195	gcaataacaggtgcttactttgctaccctgcaaaatatcct
cmp196	catatacatcctcctccatttgtatgaattcaagctttgcagg
cmp197	acaaatggaggaggatgtatatg
cmp198	aaagaaagcgatccagatgt
cmp199	acatctggatcgctttctttctttttcatccaatgttgctg
pMutin2-F	gagtgtgttgatagtgcagtatc
pMutin2-R	ctacattccctttagtaacgtgtaac
sacA-lux-back-F	tgcagtccggcaaaaaagggc
sacA-lux-back-R	ccaacttaatatcatcggtaggcc
sacA-lux-check-F	ctgattggcatggcgattg
sacA-lux-check-R	gtcgatattatgaatatcgtgccc
sacA-lux-front-F	gacagcacatgaccaggagc
sacA-lux-front-R	gttacacgttactaaagggaatgtagccaaaatcgtccagcccgttg
sacA-lux-PyhaM-F	gatactgcactatcaacacactcgtatgatttatctgaaggcggtagg
sacA-lux-PyhaM-R	gcccttttttgccggactgcacataataccagtatatcacataccgattttc

Strain WY002 [*sacA*::(P*yhaM-luxABCDE erm*) in the KU302 background] was constructed as follows. KU302 was transformed to be chloramphenicol-resistant with DNA of the plasmid pBS3Clux [27], which was linearized by digestion at the unique *Sca*I site; the resulting transformant was designated as YG010 [*sacA*::(l*uxABCDE cat*)]. The following four PCR fragments were prepared: fragment 1 corresponding to the former part of *sacA* amplified from pBS3Clux by PCR with the primer pair sacA-lux-check-F/sacA-lux-front-R (Table 2); fragment 2 for *erm* was from pMutin2 with pMutin2-F/pMutin2-R (Table 2); fragment 3 for P*yhaM* was from the chromosome of 168 with sacA-lux-PyhaM-F/sacA-lux-PyhaM-R (Table 2); and fragment 4 corresponding to the former part of *luxA* was from pBS3Clux with sacA-lux-back-F/sacA-lux-check-R (Table 2). Note that sacA-lux-front-R, sacA-lux-PyhaM-F, and sacA-lux-PyhaM-R were given complementary sequences to pMutin2-R, pMutin2-F, and sacA-lux-back-F, respectively, and the four fragments were ligated in the order from 1 to 4 by recombinant PCR using the primer pair sacA-lux-front-F/sacA-lux-back-R (Table 2). Then, YG010 was transformed to be erythromycin-resistant with the recombinant PCR fragment to express the *luxABCDE* operon under the constitutive promoter (P*yhaM*). The resulting transformant was designated as strain WY002. WY002 was further transformed to be spectinomycin-resistant with DNA of KW012 to yield KW028.

### Determination of NADPH concentrations

Two strains, 168 and KW012, were grown in 4% Soytone medium at 37 °C with shaking for 24 h. The intracellular NADPH concentration was determined in the collected cells using the EnzyChrom™ NADP/NADPH Assay Kit as instructed by the supplier (MEDIBENA Life Science & Diagnostic Solutions, Vienna, Austria). The intracellular concentration of NADPH was calculated based on the assumption that 1 OD_600_ unit corresponded to 10^9^ cells and that each cell had a volume of 1.41 μm^3^ [35].

### Conversion of inositol stereoisomers

Two strains, KU302 and KW018, were grown in 4% Soytone medium supplemented with 50 g/L *myo*-inositol at 37 °C with shaking for 24 h. Culture supernatants samples were collected at 8 and 24 h and analyzed by HPLC to quantify the concentration of *scyllo*-inositol, as described previously [10, 11] (Fig. 3B).

### Luciferase assays

Strains of *B. subtilis* were grown on LB plates at 37 °C overnight. Cells in freshly formed colonies were inoculated into 5 mL of LB medium to prepare a solution with an OD_600_ of 0.05 and allowed to grow for 4 h at 37 °C with shaking at 180 rpm. The LB cultures were diluted in 4% Soytone medium to make an OD_600_ of 0.05, and aliquots (150 μL each) were dispensed into a 96-well microtiter plate, which is set in Cytation™ 5 Cell Imaging Multi-Mode Reader (Agilent, Santa Clara, CA). The bacteria were incubated at 37 °C for 24 h with shaking. During growth, the luminescence of luciferase and OD_600_ in each well were monitored simultaneously and continuously. The luminescence [relative light unit (RLU)] was normalized by OD_600_ and expressed as RLU/OD_600_ (Fig. 4B).

The luminescence intensity of colonies was quantified as follows. Strains of *B. subtilis* were grown at 37 °C with shaking at 180 rpm for 4 h. The liquid culture was diluted 10^6^ times, and 1 mL of the dilution was spread on a plate of 4% Soytone medium agar in a rectangular petri dish (140 mm × 100 mm × 14.5 mm) and incubated at 37 °C for 11 h to form 500–1,000 colonies per plate. The plate was set in a ChemiDoc XRS + (Bio-Rad, Hercules, CA) under Chemi Hi Resolution conditions with a fixed exposure time of 90 s to capture the reversal image of the entire plate for luminescence (Fig. 6A, B). The images were analyzed using ImageJ [36] to quantify luminescence intensity (integrated density) for each colony. Under the conditions mentioned above, even the areas without colonies resulted in a luminescence intensity of up to 0.18. Thus, colonies with a luminescence intensity of 0.18 or higher were counted for histogram analysis in a window of values of 0.46 (Figs. 6C and 5D).

### Western blotting

Three strains of *B. subtilis*, KU302, WY002, and KW028, were grown in 4% Soytone medium at 37 °C with shaking for 24 h. Cells of 1 OD_600_ unit were harvested every 2 h and subjected to 12% SDS-PAGE to separate the proteins contained in the cell. The separated proteins in the gel were transferred electrically onto a PVDF membrane and soaked in TBST buffer [20 mM Tris (pH 7.4), 150 mM NaCl, and 0.05% Tween-20] containing 5% skim milk at 4 °C overnight with gentle shaking. Custom-made rabbit anti-LuxB polyclonal antibody (Sigma Aldrich, St. Louis, MO) was added and then diluted 1000-fold and allowed to react at room temperature for 1 h. The membrane was washed three times in TBST buffer for 15 min at room temperature with gentle shaking. Rabbit IgG secondary antibody peroxidase-conjugated, goat polyclonal (Rockland Immunochemicals, Pottstown, PA) was added, diluted 1000-fold, and allowed to react at room temperature for 1 h. The membrane was washed three times TBST buffer for 15 min with gentle shaking. Finally, TrueBlot: Anti-lgG HRP (Rockland Immunochemicals) was applied to the membrane to generate chemiluminescence, and then detected by ChemiDoc XRS+ (Fig. 5B).

## Data Availability

The datasets and materials used during the current study are available from the corresponding author on reasonable request.

## Abbreviations

Iol: Inositol
OD: Optical density
RLU: Relative light unit
